# The Role of Replication-Associated Repair Factors on R-Loops

**DOI:** 10.3390/genes8070171

**Published:** 2017-06-27

**Authors:** Vaibhav Bhatia, Emilia Herrera-Moyano, Andrés Aguilera, Belén Gómez-González

**Affiliations:** Andalusian Center for Molecular Biology and Regenerative Medicine-CABIMER, Universidad de Sevilla-CSIC-Universidad Pablo de Olavide, Av. Américo Vespucio 24, 41092 Seville, Spain; vaibhav.bhatia@cabimer.es (V.B.); e.herrera-moyano@lms.mrc.ac.uk (E.H.-M.); aguilo@us.es (A.A.)

**Keywords:** genetic instability, DNA-RNA hybrids, Fanconi anemia, BRCA, replication stress, cancer

## Abstract

The nascent RNA can reinvade the DNA double helix to form a structure termed the R-loop, where a single-stranded DNA (ssDNA) is accompanied by a DNA-RNA hybrid. Unresolved R-loops can impede transcription and replication processes and lead to genomic instability by a mechanism still not fully understood. In this sense, a connection between R-loops and certain chromatin markers has been reported that might play a key role in R-loop homeostasis and genome instability. To counteract the potential harmful effect of R-loops, different conserved messenger ribonucleoprotein (mRNP) biogenesis and nuclear export factors prevent R-loop formation, while ubiquitously-expressed specific ribonucleases and DNA-RNA helicases resolve DNA-RNA hybrids. However, the molecular events associated with R-loop sensing and processing are not yet known. Given that R-loops hinder replication progression, it is plausible that some DNA replication-associated factors contribute to dissolve R-loops or prevent R-loop mediated genome instability. In support of this, R-loops accumulate in cells depleted of the BRCA1, BRCA2 or the Fanconi anemia (FA) DNA repair factors, indicating that they play an active role in R-loop dissolution. In light of these results, we review our current view of the role of replication-associated DNA repair pathways in preventing the harmful consequences of R-loops.

## 1. Introduction

Soon after the discovery of the DNA double helix, it was demonstrated that the RNA strand could hybridize in vitro with single-stranded DNA (ssDNA) [1] or with double-stranded DNA (dsDNA), after its mild denaturation [2,3]. The resulting structures formed by a DNA-RNA hybrid and the displaced ssDNA were called R-loops. Now, we know that transient in vivo formation of DNA-RNA hybrids is central to many cellular processes like immunoglobulin class switching, mitochondrial replication, some transcription steps or telomere homeostasis [4,5]. Under physiological conditions, R-loops have been detected in human genes over unmethylated CpG island promoters [6], at G-rich terminator elements [7] and a number of chromosomal regions in yeast and human cells, although whether they have a positive physiological role or not is yet unknown [8,9]. However, un-programmed R-loops constitute obstacles to incoming replication forks (RFs) [10,11,12,13,14,15,16]. Consistently, R-loops can cause genomic instability in the form of hyper-recombination and chromosome fragility [4,5,14,17,18,19,20]. The formation of co-transcriptional R-loops has been inferred by different methods in yeast, *Caenorhabditis elegans* and human cells in the absence of certain RNA-binding factors [21,22,23,24,25,26,27,28].

In vivo DNA-RNA hybrids were genetically inferred in *Escherichia coli topA* mutants by suppression of their effect on ribosomal DNA (rDNA) gene expression by RNase H1 overexpression, which specifically degrades the RNA that is hybridized with DNA [29]; in yeast, by the combined use of a hammerhead ribozyme and RNase H1 [22]; and in the immunoglobulin switch regions of human lymphocytes by the bisulfite modification assay, which mutates the displaced ssDNA [30]. Molecularly, they were detected in vivo after the specific treatment of nucleic acid extracts with RNase A, which degrades free RNA and has no activity on RNA forming DNA-RNA hybrids with or without RNase H1, which specifically degrades the RNA strand of DNA-RNA hybrids, followed by a treatment with DNAse I [22]. The use of the human activation-induced cytidine deaminase (AID), an enzyme involved in immunoglobulin class switch recombination and somatic hypermutation that specifically mutates the displaced ssDNA, resulted in an additional genetic tool for the detection of R-loops [31]. The suppression of genetic instability phenotypes such as hyper-recombination or accumulation of repair foci by RNase H1 overexpression has been widely used as a genetic tool to infer the presence of R-loops in yeast, *C. elegans* and human cells. More recently, the use of the S9.6 hybrid-specific antibody allowed the direct detection of R-loops by DNA-RNA immunoprecipitation (DRIP) or immunofluorescence (IF) methods, which are currently widely used (reviewed in [32]). In addition, DNA-RNA hybrids have been detected with a tagged RNase H1 lacking its mitochondrial localization signal and a GFP-tagged RNA-DNA hybrid binding domain of RNase H1, which binds to and stabilizes RNA-DNA hybrids [6,33].

Elements like negative supercoiling or secondary RNA structures such as G quadruplexes in the non-template strand or even single- and double-stranded breaks (DSBs) can induce R-loop formation and stability (reviewed in [34]). The current model assumes that DNA-RNA hybridization is favored by retention of the nascent RNA in proximity to the negatively supercoiled DNA behind a moving RNA polymerase. This, together with the possibility that R-loops may alter the epigenetic profile of chromatin, makes R-loop resolution an intricate process. Recent reviews have extensively addressed the basic determinants of co-transcriptional R-loop formation [4,35], the possible sources and outcomes of transcription-replication conflicts, whether or not related to R-loops [18,36,37], and the different DNA repair mechanisms that have been connected to R-loop resolution [34,38]. Here, we review the recent literature about R-loop formation and resolution, focusing on understanding the impact that replication and replication-associated factors might have on R-loops.

## 2. Preventing R-Loop Formation and Accumulation

**Figure 1:**
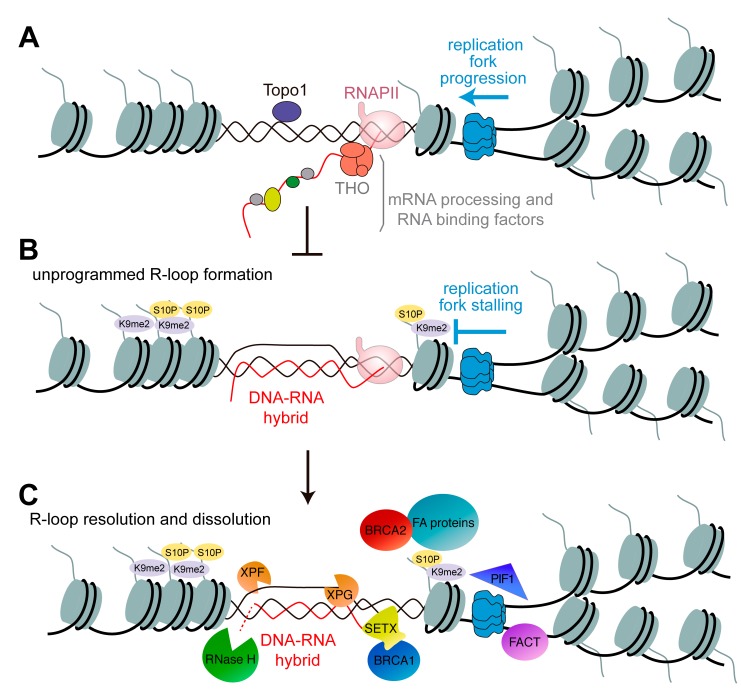
Multiple factors either prevent the accumulation of harmful R-loops or contribute to their resolution and dissolution. (**A**) The alleviation of negative supercoiling by topoisomerase I (TopoI) together with the cotranscriptional processes of messenger ribonucleoprotein (mRNP) biogenesis and several RNA binding factors contribute to preventing DNA-RNA hybridization. (**B**) In the absence of either of these factors, the nascent RNA can reinvade the DNA double helix to form a structure termed the R-loop, where a single-stranded (ssDNA) is accompanied by a DNA-RNA hybrid. R-loops and/or their associated chromatin alterations constitute a roadblock for incoming replication forks. (**C**) Ribonuclease (RNase) H and helicases, such as Senataxin (SETX), control R-loop levels in the cells by degrading the RNA moiety of the R-loops or unwinding DNA-RNA hybrids, respectively. BRCA1 recruits SETX to R-loop sites. FACT, PIF1, BRCA2 and Fanconi anemia (FA) factors help the replisome to overcome R-loops. XPG and XPF nucleases can also target R-loops, thus generating DNA breaks.

Transcription, messenger ribonucleoprotein (mRNP) biogenesis and export have evolved as coupled processes in eukaryotes to the extent that protein complexes responsible for distinct steps during mRNP biogenesis and export share common subunit proteins [39]. This sequential coupling is important in preventing accumulation of co-transcriptional R-loops [40] (Figure 1A). Depletion of some of those complexes may lead to nascent RNA retention at the transcription site and promote R-loop accumulation [21,22,23,25,26,27,28]. A long-established example is the transcription-associated THO complex, the absence of which, leads to transcription elongation impairment and spontaneous hyper-recombination in yeast [41,42,43,44,45] and human cells [46]. The presence of R-loops has been confirmed in human cells depleted of several other mRNP processing, helicases and RNA binding factors, including THSC/TREX-2, SRSF1, DDX1, DDX19, DDX23, SETX/Sen1, AQR, XRN2 and others [7,20,23,24,25,26,27,28,33,47,48,49]. The importance of such mechanisms that control R-loop levels in cells is highlighted by the fact that the removal of those factors leads to genome instability and replication stress, which are common features found in pre-cancerous and cancerous cells (reviewed in [50]).

Co-transcriptional R-loops can also form in wild-type cells and might have regulatory roles. DRIP-chip data in *Saccharomyces cerevisiae* cells demonstrated a prevalence of DNA-RNA hybrids at the rDNA locus, telomeric repeat regions, highly transcribed regions and antisense-associated genes [51]. A recent study using an additional step of nuclease S1 digestions has expanded the regions able to form R-loops to some AT-rich sequences, which leaves several open questions about the methods of detection and the way R-loops are formed or stabilized [8]. In human cells, DRIP coupled with deep sequencing (DRIP-seq) data pointed to CpG island promoters and transcription termination regions with high GC skew, regions with rich purine skew at the RNA-coding strand, rDNA and satellite repeats as the main genomic sites for R-loop accumulation [6,52,53].

Finally, chromatin state is emerging as a putative important element influencing R-loop accumulation. Thus, in *C. elegans*, histone H3 lysine 9 di- and tri-methylations (H3K9me2 and me3), chromatin marks that are normally associated with heterochromatin, have been implicated in the prevention of DNA-RNA hybrid formation [54]. Furthermore, topoisomerase inhibitors promote both chromatin decondensation and R-loop formation [55]. These observations suggested that R-loop formation is directly influenced by the chromatin state. A more opened chromatin could favor R-loop formation by increasing the chances of nascent DNA-RNA hybridization. Supporting this, deletion of the SNF2-like nucleosome-remodeling factor Fft3 in *Schizosaccharomyces pombe*, which causes an enhanced nucleosome turnover, can also promote the formation of RNA-DNA hybrids [56]. R-loop formation can also be influenced by chromatin in an indirect manner. For example, histone H3 arginine 17 and histone H4 arginine 3 dimethyl asymmetric (H3R17me2a and H4R3me2a) marks serve to recruit the TDRD3-TOP3B protein complex and dampen the negative supercoiling and its consequent R-loop formation [57]. This is an important focus of research that should provide important clues to understand how R-loop formation is facilitated.

## 3. Impact of R-Loops on Chromatin Structure

Different studies in *S. cerevisiae*, *S. pombe*, *C. elegans* and human cells initially showed an association between R-loops and chromatin modifications with specific physiological consequences [6,58,59,60]. From global genome studies, we now know that the presence of R-loops in human cells correlates with certain epigenomic signatures, such as methylated histone H3 (H3K4me1, H3K4me2, H3K36me3), as well as acetylated histone H3 at promoter regions or the enhancer- and insulator-like state at terminators [6,9,39]. Nevertheless, the cause and effect relationship between R-loop accumulation and R-loop-associated chromatin marks is unclear. Indeed, several studies suggest that, once formed, R-loops can affect chromatin (Figure 1B). Thus, RNAi-mediated transcriptional gene silencing at centromeric regions, which involves the accumulation of the H3K9me2 repressive marker, is influenced by the levels of DNA-RNA hybrid formation in *S. pombe* [58]. R-loops produced in the absence of the THO complex in yeast, human cells or *C. elegans* trigger an accumulation of histone H3 serine 10 phosphorylation (H3S10-P) [59]. H3S10-P is a chromatin modification normally associated with chromatin condensation, which can indeed cause chromosome fragility [61]. In addition, high levels of H3K9me2 were observed in the absence of the THO complex in *C. elegans* and human cells [59], and R-loops form and colocalize with H3K9me2 and trigger silencing at the expanded repeats in Friedreich ataxia (FRDA) and fragile X syndrome (FXS) patients [62,63,64]. Along the same line, R-loops trigger antisense transcription over G-rich terminator elements leading to an increase of H3K9me2 together with recruitment of the heterochromatin protein 1γ (HP1γ) to facilitate transcription termination [60].

We do not know the mechanism by which R-loops might influence chromatin structure. However, evidence suggests that R-loops can indirectly affect chromatin by modulating the binding of chromatin-regulatory complexes, as seen for Tip60-p400 and PRC2 in embryonic stem cells [65], or the recruitment of histone variants, as seen for histone H3.3 [66]. Altogether, these observations support that R-loops can directly or indirectly influence the chromatin state and raise the question of whether chromatin alterations might be a major responsible for the role of R-loops in different cellular processes, such as transcription, DNA replication or DNA repair, thus affecting their impact on genome integrity. Importantly, we have just reported yeast histone mutants that favor the accumulation of R-loops without leading to genome instability, implying that R-loops do not cause genome instability by themselves [67]. Instead, R-loop-driven chromatin alterations, such as H3S10-P, are a contributor of genome instability, suggesting a two-step mechanism in which, first, an altered chromatin facilitates R-loops; and second, chromatin is modified compromising genome integrity [67].

## 4. DNA Replication and Repair in the Context of R-Loops

R-loop-induced DSB formation has been shown to require transcription-coupled nucleotide excision repair (NER) factors suggesting that NER may also be a clearance pathway for naturally-occurring R-loops [68] (Figure 1C). Nevertheless, the major mechanism by which R-loop accumulation is known to trigger genome instability is by perturbing DNA replication [10,11,12,13,14,15,16,21,69,70]. Despite the compelling evidence supporting that R-loops constitute a barrier for RF progression, the mechanism is still unknown. In light of the connection between R-loops and the chromatin state discussed above and the recent observation that R-loop-mediated chromatin alterations are required to compromise genome stability [67], it would be interesting to explore R-loop-induced heterochromatinization as a putative key intermediate for R-loop-mediated replication impairment and the consequent genome instability. Among other possible mechanisms proposed to explain R-loop-dependent genetic instability, it is noteworthy that DNA-RNA hybrids can promote unscheduled origin-independent replication priming events, as has been observed in the rDNA [71], which may increase the probability of replication-transcription encounters. Regardless of the mechanism, RF collapse would explain the R loop-associated genetic instability phenotypes, such as DNA breaks and hyper-recombination.

It is noteworthy that the accumulation of RNA-DNA hybrids could also affect the repair of DSBs that may form after RF collapse. Two recent reports showed that DNA-RNA hybrids form at I-*Sce*I- and I-*Ppo*I-induced DSBs in human and *S. pombe* cells, respectively, by the hybridization of the RNA with the resected ssDNA, thus limiting the post-resection steps to occur [48,72]. In human cells, the DDX1 RNA helicase forms foci after ionizing radiation (IR) treatment, and it is required to clear DNA-RNA hybrids and allow homologous recombination (HR) to proceed [48]. In yeast cells, an equilibrated activity of RNase H is required to promote replication protein A (RPA) recruitment and HR repair [72]. These reports open a new view of the role, whether positive or negative, that DNA breaks could have in DNA repair, which has been discussed in a different review [34].

Given that transcription seems a frequent lesion faced by RFs [73], the DNA-RNA helicase Senataxin (SETX), which is involved in the removal of DNA-RNA hybrids during transcription termination, associates also with RFs to protect their integrity across transcribed genes [69]. At the same time, it is conceivable that some replisome-associated factors would promote replication through R-loop-containing regions, thus aiding in R-loop removal and preventing genetic instability (Figure 1C). In this regard, the key sensor of ssDNA at stalled RFs, RPA, has recently been reported to sense R-loops and regulate RNaseH1 action [74]. Furthermore, the histone chaperone complex FACT, required for replication through chromatinized DNA, as recently demonstrated in vitro [75], prevents R-loop-mediated genome instability in yeast and human cells [76]. Furthermore, R-loops accumulate in human cells depleted of DNA repair factors that act during replication, such as those involved in the FA/BRCA pathway [77], that is in the tumor suppressor and breast cancer-susceptibility genes BRCA1 and BRCA2 [33,78] or the Fanconi anemia (FA) factors FANCD2, FANCA or FANCM [14,79]. Last, a recent report in yeast has shown an RNaseH-sensitive increase in recombination and gross chromosomal rearrangements at the tRNA genes in the absence of Pif1 and the replisome component Rrm3 [80], suggesting a role for both helicases (yeast homologs of the human PIF1) in the avoidance of R-loop-dependent genetic instability.

In summary, evidence suggests that there are a number of potential players in the mechanism by which RF progression may trigger genome instability at R-loops. However, an important body of experiments at both the genetic and molecular level is still required to understand this phenomenon.

## 5. Role of BRCA Genes in R-Loop Resolution

Mutations in BRCA1 and 2 increase susceptibility to breast, ovarian, pancreatic, prostate cancers and also lead to FA [81]. Pan-nuclear staining of γ-H2AX, a marker for replication stress, was observed in Capan-1 BRCA2-deficient cells and disappears after overexpression of RNase H1 [33]. Moreover, R-loop stabilization correlates with adhesion-independent proliferation in retinal pigment epithelial cells RPE-1, a hallmark of cell transformation [33]. Since replicative stress is the main cause of tumorigenesis, the cancer susceptibility phenotypes conferred by BRCA deficiency could be in part attributed to the inefficient removal of co-transcriptionally-formed R-loops. In this regard, the frequency of somatic mutations in both BRCA1 and BRCA2 mutant cancer genomes increases with the distance from the transcription start sites [82]. This might reflect R-loop formation because the accumulation of transcription-associated negative supercoiling, which contributes to R-loop accumulation, increases along the genes’ body [15,29,83,84]. In addition, C to T and C to A changes are frequent in BRCA-mutated breast cancer cells [82]. Thus, cytosine deaminase enzymes targeting the unprotected ssDNA from the R-loop, such as AID, could cause the C to T hypermutation.

The action of BRCA1 is localized to R-loop-rich transcription termination regions, where it mediates SETX recruitment [78,85]. Consistently, the genomes of BRCA1-mutant breast cancer patients show frequent insertions and deletions in R-loop-rich transcription termination regions [78], whereas these mutations appear throughout the genome in BRCA2-mutated breast cancers [78,82]. Thus, it is still an open question if the functions of BRCA1 and BRCA2 in R-loop removal are inter-dependent and whether their roles are connected to their known functions in DSB repair.

BRCA2 is a multi-domain protein crucial for multiple cellular functions, such as HR, by promoting RAD51 loading [86], interstrand crosslink (ICL) repair [87], RF stability [88,89] and cytokinesis [90,91]. Therefore, the accumulation of R-loops in BRCA2-depleted cells might be explained in several ways. Elucidating which of the BRCA2 domains and functions is behind the resolution of R-loops will be an important task for the future. BRCA2 is stabilized by the DSS1 component of the THSC/TREX-2 RNA export complex [92] and interacts with another component of TREX-2, PCID2 [33], although the nature and timing of such interactions are unclear. TREX-2 could affect the stability, as well as the cellular localization of BRCA2, and indeed, BRCA2 loading onto chromatin is partially relieved by the presence of RNase H1 [33]. Possibly, BRCA2 helps prevent or resolve R-loops by an on-site action, either directly or indirectly by recruiting other factors involved in R-loop processing. Given that R-loop accumulation has not been detected upon depletion of the HR protein Rad51 [33], it could be possible that the main role of BRCA2 in R-loop dissolution was not related to its DSB repair function. BRCA2’s role in R-loop dissolution could be linked to its role in the stability of RFs since not only BRCA2 (FANCD1), but also BRCA1 (FANCS) and FANCD2, all of which are FA proteins, are involved in avoiding excessive MRE11-mediated fork degradation and the consequent genome instability [88,93,94,95].

## 6. Role of the Fanconi Anemia Repair Pathway in R-Loop Resolution

The resolution of R-loops depends on components of the FA pathway including BRCA2, BRCA1, FANCD2, FANCA and FANCM [14,79]. The FA pathway is important for the repair of ICLs, which are complex and cytotoxic lesions able to impair RF progression. Consistently, the action of the FA pathway is induced only during DNA replication, via a tight regulation of FANCD2 monoubiquitination [96]. The widely-accepted model states that when an RF encounters an ICL, the FA-pathway is activated by phosphorylation and ubiquitination of FANCI-FANCD2 by ATR and the FA-core complex, respectively. FANCD2 regulates the action of SLX4, a scaffold protein that recruits XPF, MUS81 and SLX1 endonucleases. The nuclease action generates a gap of ssDNA and a dsDNA break, which are repaired by trans lesion synthesis (TLS) and HR, respectively [97,98].

Cells from FA patients show enhanced sensitivity to ICLs, but also spontaneous chromosome breakage, suggesting that this pathway is not only required for ICL repair, but also essential in resolving endogenous DNA damage [96]. Among the sources of this endogenous DNA damage are R-loops. In support of this, human and murine cells defective in FANCD2 or FANCA accumulate R-loops as shown by DRIP assays, and a large proportion of the DNA breaks in FA cells and FANCD2 foci in HeLa cells are R-loop dependent [79]. Thus, apart from its function in ICL repair, the FA pathway is also activated in response to replication stress, FANCD2 protein being implicated, as BRCA1 and BRCA2, in the stabilization of stalled RFs [94,99]. FA proteins, through FANCM translocase activity, help to avoid R-loop-mediated arrest of RFs as determined using DNA combing and DRIP [14].

The importance of the FA pathway in RF stability and progression after encountering R-loops is also highlighted by the recent finding that R-loops are the main cause of RF arrest at fragile site regions in *FANCD2*-/- patient-derived lymphoblast, although FANCD2 ubiquitination seems to be dispensable for this function [19]. In this study, R-loops were observed to accumulate at the common fragile site (CFS) CFS-FRA16D in *FANCD2*-/- lymphoblast cells and single-molecule analysis of replicated DNA (SMARD) showed that the elimination of DNA-RNA hybrids suppresses the replication perturbations observed in this CFS region [19].

We believe that as for ICL, FA core proteins could orchestrate the action of multiple DNA repair pathways, such as helicases or nucleases, for R-loop dissolution. BRCA2 could scan and recognize the R-loops and the associated signatures like ssDNA or ssDNA-dsDNA interface, thus having a role upstream in the FA pathway. In agreement, BRCA2 interacts with basal levels of ubiquitinated-FANCD2 even in the absence of any external DNA damage in HeLa cells [100]. In addition, XPF and XPG NER endonucleases may work to create ssDNA breaks on the flanking DNA of stable R-loops [68] that may be finally converted into DSBs during DNA replication and repaired by HR.

## 7. Concluding Remarks

We now know that factors known to work at replication, such as FA/BRCA and FACT complexes, assist and protect RFs at R-loop-enriched regions. These complexes work together with the DNA-RNA hybrid specific enzymes SETX, RNase H1, etc., and the numerous mRNP-associated factors, such as THO, SRSF1 or some DDX proteins, to prevent R-loop-mediated genome instability. A recent burst of literature on new factors whose depletion causes the accumulation of R-loops together with the longstanding observations that transcription and R-loops threaten genetic stability claim R-loops as a main source of genetic instability. Given that genetic instability is a hallmark of tumor cells, this opens the possibility that R-loops are oncogenic structures that could not only be used in diagnosis, but also explored as a potential therapeutic target. The recent findings that R-loops are behind certain human syndromes, such as Aicardi-Goutières syndrome (AGS) [101] or immunodeficiency, centromeric instability and facial anomalies (ICF) syndrome [102], further highlight the relevance of R-loop prevention and resolution in human health. Future efforts aimed at finding new factors involved in R-loop prevention, deciphering the role of RFs dealing with DNA-RNA hybrids and the implication of chromatin modifications in R-loop-mediated genome instability will help to gain insight into the mechanisms that cells use in the resolution of R-loops.
